# Progress in Inherited Retinal Disease Drug Discovery and Development: A Foundation’s Perspective

**DOI:** 10.1007/s11095-018-2514-2

**Published:** 2018-10-18

**Authors:** Benjamin Yerxa

**Affiliations:** Foundation Fighting Blindness, 7168 Columbia Gateway Drive, Suite 100, Columbia, Maryland 21046 USA

**Keywords:** inherited retinal degenerative disease

## Abstract

Ophthalmic drug discovery and development has enjoyed a recent renaissance, with a major shift away from reformulating old systemic drugs for ocular use to de novo discovery of drugs for specific ocular disease targets. This shift, coupled with a revolution in molecular biology and genetic sequencing, has uncovered an unprecedented number and variety of novel targets for therapeutic intervention in eye disease. With such a treasure chest of new science to pursue, it also creates a new challenge for translating the lab-based discoveries through the translational “valley of death” into full scale industry-led development of new, approved therapeutics to treat eye disease. This is in fact a daunting task, as the cost of drug development continues to increase and many of the new therapeutic targets are based on smaller, orphan diseases with very high unmet medical needs. This perspective focuses on the role of a nonprofit foundation, The Foundation Fighting Blindness, in fueling and supporting the advancement of new therapies for blinding inherited retinal degenerative diseases into approved therapeutics. The new collaborative model is changing the way breakthrough drugs are coming to market for patients, and innovative funding models are required to match the innovative science.

In order to understand where the inherited retinal degenerative disease (IRD) field is today, it is useful to quickly revisit the recent past. In the modern era of drug discovery, the efforts in the area of ocular research formally began about 50 years ago with the creation of the National Eye Institute (NEI) in 1968. The formation of the NEI was due in part to the urging and support of a new foundation, Research to Prevent Blindness, founded in 1960 by Dr. Jules Stein. At this time, the NEI and vision research was focused on major diseases such as cataracts which, even to this day, is still the number one cause of treatable blindness worldwide. It is no wonder then that the poorly understood orphan diseases of the retina received little initial funding from government agencies and other funding sources.

In 1971, just a few years after the NEI got its start, a new foundation was formed by a group of families, led by the Berman and Gund families, who were affected by an inherited retinal degenerative disease called retinitis pigmentosa (RP). The National Retinitis Pigmentosa Foundation was thus formed to fund one of the first retinal research laboratories in the US: Dr. Eliot Berson’s lab at Massachusetts Eye and Ear at Harvard Medical School. Imagine that during this era when an individual or family was diagnosed with an IRD like RP they were told that the cause of the disease was unknown, there is nothing that can be done, and to prepare for going blind. The brave families that started this foundation (now named the Foundation Fighting Blindness) decided to fight this unacceptable news and started funding the smartest minds in science to figure it out.

Now let’s fast forward to today, when we know that the group of retinal diseases called IRDs comprises dozens of different, predominantly monogenic, orphan retinal diseases, both syndromic and nonsyndromic, includes over 200,000 patients in the US alone and has over 250 known disease-causing genes. When we include age-related macular degeneration (AMD), where the multiple genetic drivers of disease are just beginning to be uncovered, these patient numbers skyrocket to 10 million in the US alone. The spectrum of IRDs has RP as the largest member at about 50% of IRDs, followed by Stargardt disease at about 15%, with others filling out the spectrum: retinoschisis, choroideremia, achromatopsia, congenital stationary night blindness, BEST disease and many more [1]. Each of these named diseases hide a complexity of genetic subtypes, for example RP alone can be caused by a mutation in any one of at least 87 genes and similar complexity is seen within subtypes of Leber congenital amaurosis (LCA), Usher syndrome and other IRDs. Thus, at a genetic level, many of these specific IRDs are exceedingly rare, with only a few tens to hundreds of patients in the US. IRDs affect all races and geographies with some demographic concentrations, such as the EYS form of RP in Japan [2].

Today, the Foundation Fighting Blindness funds over 90 investigators around the world, many of whom are early in their careers and in need of leverage to garner larger NIH grants. The Foundation Fighting Blindness has historically funded young investigators to stoke a career-long interest in finding treatments for retinal diseases. Clinicians are often under pressure to move away from IRDs due to the much larger time commitment required per patient visit. Career Development Awards from the Foundation Fighting Blindness can also help alleviate some of the financial stress that comes early in a physician’s career while they are establishing their leadership in the field. Attracting key talent and retaining their interest for careers in retina and IRDs is a key role for the Foundation Fighting Blindness and arguably one of the most effective things it does.

A big part of the deep understanding of the IRDs is due to the revolution in molecular biology and genetic sequencing that started with the 13 year effort to complete the sequencing of the human genome in April, 2003 at a cost of about $1 billion. Looking at the cost and speed of genetic testing over the last several decades, it wasn’t until the last 10–15 years that sequencing became truly automated, fast and relatively affordable. The biggest jump in sequencing productivity occurred in 2005 with the advent of the Solexa/Illumina sequencer, leading to an eight order of magnitude increase in output [3]. Now, individuals can have all known IRD-related genes and mutations analyzed in a few weeks for a few thousand dollars. Although many IRD disease-causing genes remain elusive still, it is now possible to identify and understand disease-causing genetic mutations in many individuals on a routine basis.

Because of these tremendous advances, patient registries and genetic testing have become an area for foundations such as the Foundation Fighting Blindness to play a role. For example, the Foundation Fighting Blindness has established a patient registry, called My Retina Tracker®, which allows individual patients to securely enter and control their own information and the ability for their physician to add additional medical data. The Foundation Fighting Blindness has limited funding to provide free genetic testing and counseling for those who participate in My Retina Tracker so that the genetic data can be added to enhance the registry. As the registry grows over time, now with over 8000 user profiles, this database of information can be used to help better understand the prevalence of specific genetic IRDs and match patients up with clinical trials or new therapies as they are developed and approved.

One of the roles of foundations such as The Foundation Fighting Blindness is, from time to time, to take a chance on sponsoring a clinical trial of a therapeutic agent. An example of this is a study that The Foundation Fighting Blindness sponsored using valproic acid (VPA), a drug with reported effects on retinal neurotrophic and growth factors, for treating autosomal dominant RP that followed 90 patients for 12 months [4]. While the VPA study showed no efficacy, the untreated control arm of the study became a useful natural history study of RP, and as such, was the largest of its type ever performed. The silver lining was that further analysis of this well-controlled data-set led to the discovery of a new anatomical endpoint, called EZ area, in which the loss of photoreceptors over time was quantified by optical coherence tomography (OCT) measurements [5]. This finding resulted in the submission of a full briefing package to the Food and Drug Administration (FDA) and a workshop with the FDA to review and discuss novel endpoints like EZ area, ultimately resulting in a publication with the FDA and the NEI [6]. Having an objective, anatomical endpoint that is acceptable, in principle, by the FDA for a slowly degenerative disease was a huge breakthrough for the field, and this endpoint is now incorporated into many industry sponsored clinical protocols that study therapeutic approaches to IRDs.

Having a clear understanding of the natural history of a disease, especially for a rare degenerative disease, is very important. As orphan indications, the patient populations are very limited. Having an adequate and well-controlled natural history study that gets published in a peer reviewed journal can help with new protocol designs, defining patient populations, statistical powering and determining early efficacy as proof of concept. Although the FDA is likely to require a control group as part of a pivotal trial, having the natural course of the disease quantified ahead of time allows for companies to get an idea of potential benefit early on in development and optimize the length of the study to observe a statistically significant outcome. Thus the conduct of natural history studies has emerged as a key role for the Foundation Fighting Blindness to spur further investment in IRD therapies.

An example of this kind of work is the recently completed ProgStar study that evaluated the natural history of Stargardt disease. ProgStar was run as an academic consortium funded by the Foundation Fighting Blindness to provide a deep understanding of the natural history of Stargardt disease. The study evaluated 460 eyes over two years and cost over $6 million to complete. To date, the ProgStar Study Group has published over 10 peer reviewed articles, including several that outline important potential therapeutic endpoints such as fundus autofluorescence (FAF) and segmented OCT [7]. It has also generated significant industry interest and use. To continue this kind of work, the Foundation Fighting Blindness has established a clinical consortium in collaboration with the JAEB Center for Health Research to conduct new natural history studies, such as the ongoing one in Usher syndrome 2A, called RUSH2A, which is expected to complete enrollment by the end of 2018. With many other smaller IRDs left to study, finding efficient, collaborative and affordable ways to conduct natural history studies going forward will be very important.

In the early years of funding retinal research, the Foundation Fighting Blindness and other vision funders have provided grants to researchers, departments and institutions to study the basic science of IRDs. It took decades of long-term funding to set the stage for where we are today. Now, with so much of the core disease processes understood, the funding paradigm has had to adapt to a more translational mode by funding therapeutic approaches on a milestone basis. This evolution was necessary to keep moving the pipeline of new discoveries forward and into early development all the while maintaining sufficient levels of basic research funding to fuel future discoveries. Ten years ago there were only a few IRD programs in clinical development. Now there are more than 30 active clinical development programs with only one currently funded by the Foundation Fighting Blindness, and the number of preclinical programs has grown significantly. All of this progress poses yet another set of challenges for foundations and the IRD field to adapt to.

In order to continue the pace of progress, innovation in funding models is an imperative. Drug development costs are in the tens of millions of dollars or more for each program and in reality have a very high failure rate. Thus, traditional nonprofit money-raising and grant-making, although important, is inadequate to move the field forward through clinical trials towards approval. The key is to find ways to leverage funding dollars with collaborations. The collaborations necessarily have to be with other partners in the ecosystem that are essential for seeing that new therapies actually make it to patients, such as venture capital and private equity firms, pharmaceutical and biotechnology companies, patients and families, institutional investors, other foundations, government agencies and managed care companies.

One innovation in funding R&D was pioneered by the Cystic Fibrosis (CF) Foundation over 10 years ago called venture philanthropy [8]. This risk-sharing model successfully led to the first mechanism-based therapeutic approval in the CF space while also providing long term financial resources to aggressively further its mission. Another name for this kind of funding is program-related, or mission-related, investment and has been scaled up by the Gates Foundation to further its mission where traditional grant-making is inadequate [9]. In fact, many nonprofit research funders have active venture programs of various types, such as the Juvenile Diabetes Research Foundation, Leukemia and Lymphoma Society, and others. It is no surprise then to see foundations like the Foundation Fighting Blindness stepping into this new funding paradigm with mission-related investments of its own in order to fund future therapeutics through the translational funding gap. The Foundation Fighting Blindness has recently formed a standalone, dedicated venture philanthropy investment vehicle, called the Retinal Degeneration Fund, which makes mission-related investments with any returns reinvested in its mission.

A new, innovative funding model for translational eye research has also been proposed called Eye Bonds. This pilot program, which was recently introduced in the House of Representatives as H.R. 6421, the Faster Treatments and Cures for Eye Diseases Act, proposes to leverage a limited federal guarantee to reduce investment risk and has the potential to provide up to a billion dollars of funding for translational eye research projects [10]. Such a bio bond, much like the now $200 billion green bond market, opens up another channel of funding, such as investment by pension plans and insurance companies, that would otherwise be considered too risky. The Foundation Fighting Blindness supports initiatives like Eye Bonds in order to increase overall funding for eye research.

A great success story of technical achievement and public-private partnership is the recent development and approval of the gene therapy voretigene neparvovec (LUXTURNA™) for the treatment of RPE65 LCA [11]. The story of this groundbreaking treatment began with the discovery of the gene *Rpe65* nearly 25 years ago at the NEI, and was continued by many labs around the world. The Foundation Fighting Blindness played a role by investing $10 million in studies that linked mutations in RPE65 to blindness, helping to advance this new gene therapy into clinical trials. The funding took many forms, such as funding a career development award for Dr. Jean Bennett that enabled her to obtain a larger NEI grant to pursue *in vivo* gene therapy techniques, as well as funding a pivotal laboratory breakthrough from the University of Pennsylvania (Drs. Bennett, Maguire, Jacobson, Aguirre, Cideciyan), Cornell (Dr. Acland), and the University of Florida (Dr. Hauswirth) that restored vision to Briard dogs born blind from LCA. This strong proof of principle set the stage for the RPE65 gene therapy clinical trial that launched in 2007 at Children’s Hospital of Philadelphia (CHOP), led by Drs. Bennett and Maguire. In 2013 a biotechnology company, called Spark Therapeutics, was spun out of the University of Pennsylvania to bring this and other gene therapies to patients. Spark was able to raise significant capital, none of which came from the Foundation Fighting Blindness, to push this new therapy through clinical development, including the development of a novel clinical endpoint, to achieve the first US approval of a gene therapy for any inherited disease. This watershed event in December 2017 marks the beginning of a new age of IRD research and development, although there are significant unmet medical needs and knowledge gaps yet to be conquered [12].

In summary, this is an exciting time to be discovering and developing new therapies for IRDs and macular degenerations. Rapid and cost effective genetic testing is leading the way for patients and drug developers to identify and understand IRDs. The eye has become a new proving ground for innovative biotechnologies due to its small size, immune privilege, ready access and ease of observation—a small amount of medicine can go a long way, reducing manufacturing costs and clinical trial risks. The number of programs in early development has skyrocketed and we are now in the post-Luxturna age of rapid progress in gene therapies. As technical progress accelerates, foundations such as the Foundation Fighting Blindness have been adapting funding models to this changing environment in order to be the most effective at accomplishing its mission: To provide preventions, treatments and cures for inherited retinal diseases and macular degeneration as fast as possible.
